# The Redesign of a Checklist for Evaluating Driver Impairment: A Human Factors and Ergonomics Approach

**DOI:** 10.3390/healthcare10071292

**Published:** 2022-07-12

**Authors:** Tanja Baertsch, Marino Menozzi

**Affiliations:** Department of Health Sciences and Technology, ETH Zürich, 8092 Zurich, Switzerland; mmenozzi@ethz.ch

**Keywords:** checklist design, design principles, impaired driving, road safety

## Abstract

The Cantonal Police of Zurich, Switzerland, use a checklist to identify impaired drivers when conducting traffic stops. This checklist was developed by subject-matter experts and has been in use for eight years. The goal of this study was to redesign the checklist while considering human factors and ergonomics principles in combination with findings from a retrospective analysis of a set of 593 completed checklists. The checklist was amended in accordance with the results of the retrospective analysis by adding missing items and discarding superfluous ones. In addition, a hierarchical cluster analysis of the retrospective data suggested an improved spatial organization of checklist elements and the grouping of similar items of the checklist. Furthermore, aspects related to Fitts’s law, visual complexity, and an optimized direction of processing the checklist underpinned the design process. The results of an evaluation of the redesigned checklist by 11 laypeople and 13 police officers indicated an improved usability of the redesigned checklist over the original.

## 1. Introduction

Several factors have contributed to the popularity of checklists as tools in the workplace. Checklists help workers complete tasks effectively and consistently. Unsurprisingly, checklists are frequently used to assist in executing complex tasks such as preparing an airplane for a flight [1] or performing surgery [2,3,4]. Checklists are also frequently used in simple tasks, for instance, to overcome memory limitations; examples of checklists as memory aids include shopping and ‘to do’ lists. The potential benefit of a checklists depends on its design, as a poor design reduces task effectivity and efficiency and could even make the checklist irrelevant [5,6].

The development and design of checklists has been examined in the literature. In an early work, Easterby [7] suggested that a good checklist should be brief, sharp, and clear. Based on observations of flight crews in action, interviews with flight crews, and examinations of aircraft accidents, Degani et al. [8] compiled guidelines for checklist design. These included dividing long checklists into subcategories that address similar functions and placing the most critical items as close to the beginning of the checklist as possible. More recently, Burian et al. [5] introduced a framework for the systematic development of checklists involving six phases: conception, design, evaluation, use, modification, and termination. The conception phase refers to the analysis of potential concerns. In this phase, so-called subject-matter experts are the most essential contributors in identifying items to be included in the checklist. The design phase focuses on the application of human factors and ergonomics principles to the layout of the checklist with the goal of optimizing its usability and effectiveness. Many factors need to be considered when designing checklists, including layout, font, size, and navigation method, as explained by Burian et al. (see Table 1 and Figure 2 in [5]). After conception and design, the usability of the checklist is evaluated and modified before it is eventually released for application. Ideally, the phases are repeated until no further modifications are required.

**Figure 1:**
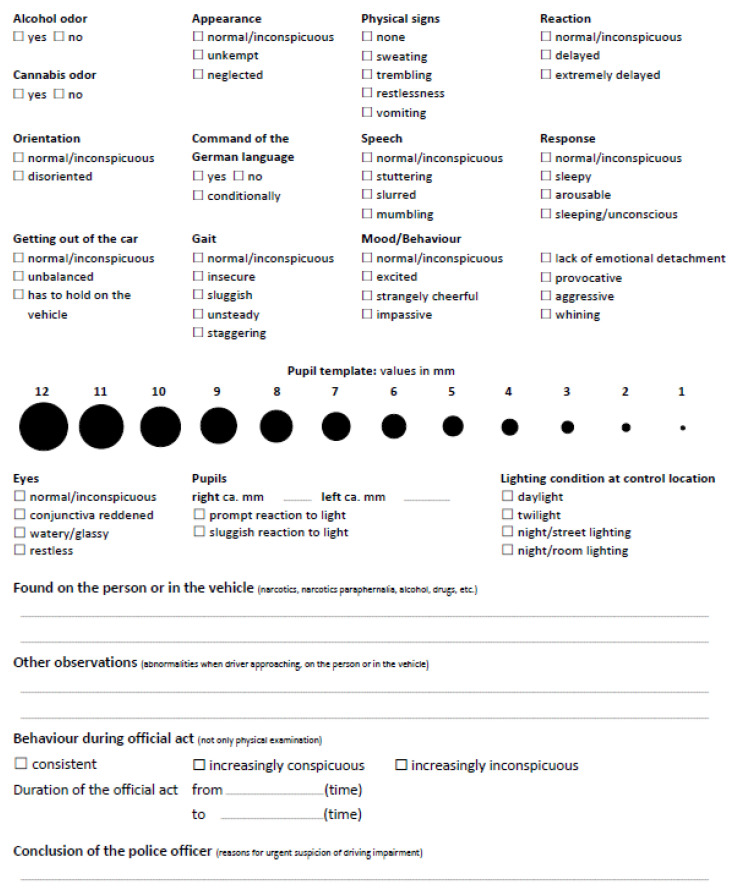
The original VERIFY checklist, translated from German.

In collaboration with the institute of forensic medicine at the local university, police officers of the Canton Zürich (Switzerland), who are experts in traffic control, created a checklist to ensure an effective, consistent identification of impaired drivers during traffic stops. The paper checklist (Figure 1), which is in a portrait format and has a size of DIN A4, consists of 16 observational items (e.g., speech, reaction, and appearance) including two or more response categories (e.g., appearance: normal, unkempt, and neglected). The two open questions in the checklist allow for the noting of observations not covered by the 16 items. The checklist is part of the observational method VERIFY (a German acronym: Verfahren zur Identifikation von Fahrunfähigkeit—Procedure for identification of driving impairment) and has been successfully utilized for traffic control since 2014. Detailed information on the theory behind VERIFY can be found in the work of Baertsch et al. [9]. Ideally, the checklist is applied immediately after drivers have been stopped by the police. Observations, as perceived by the officer, are marked on the checklist and, if there are sufficient indications of impaired driving, a prosecutor decides whether the observed indicators should constitute further legal action. As the observations have a strong influence on the prosecution process, it is essential that they are performed with great care, which can be challenging, as the completion of the checklist sometimes occurs in distraction-rich environments or stressful situations. Therefore, the checklist should be designed so that police officers can process it effectively and efficiently without having had prior experience with it.

Today, the police of the Canton Zurich have eight years of experience with the checklist, which gave reason to progress to the modification phase, being the next phase in checklist development, and to redesign the checklist considering human factors and ergonomics principles as well as the accumulated experience. The redesign process was performed with a particular emphasis on visual complexity, Gestalt principles, the horizontal/vertical arrangement of the response categories, the reading direction, and Fitts’s law regarding pointing tasks. The rationale for considering these five factors is briefly explained in the next paragraph; it is worth noting that they are related to ideas of how to improve checklists as per various guidelines, design handbooks, and the literature as reported above. The redesigned version of the checklist was evaluated by means of interviews with both laypeople and police officers.

Visual complexity affects cognitive speed and processing ability [10,11,12,13] and, therefore, affects the efficiency of a checklist. Visual complexity is also directly related to a checklist’s effectiveness because of the potential for increases in both lack of time and errors. One frequently addressed issue among the various facets of visual complexity is the quantity of information; the number of items in a list, for example [13,14]. Therefore, a checklist should consist of as few items and visual features as possible [5,7]. Furthermore, visual complexity is influenced by the variety involved in the visual qualities of the displayed objects, including colors, shapes, and sizes, and by the spatial organization of checklist elements. Structuring information in groups and separating the groups with white space can improve the clarity of the layout and make it more readable [14]. Similarly, Gestalt principles can improve the perceived structure of a checklist, reducing the difficulty and time needed to retrieve information [15,16]. Gestalt principles are organized into six global categories, namely, closure, hierarchy, prägnanz, proximity, similarity, and symmetry; only hierarchy, proximity, similarity, and symmetry were relevant here.

Checking boxes by hand requires moving the pencil from box to box; a landmark study on such types of movement was written by Fitts in 1954, leading to them being called Fitts’s tasks. Movement time [17,18] and cognitive load [19] have been shown to increase alongside the Fitts task’s index of difficulty (ID), which is a log_2_ function of the ratio between the distance between the two checkboxes (A) and the size of the checkbox (W) at the location of arrival; ID = log_2_(1 + A/W). The efficiency and usability of a checklist can be improved by reducing the ID of the pointing tasks; checkboxes should be of sufficient size and arranged such that they minimize the total travel path of the hand.

The various ways of arranging the items and response categories within a group, including vertical (item and response categories all in one column), block (two columns of response categories and the item at the beginning of the first column), and T-format (headline on top followed by two columns of response categories) affect the usability of the checklist. In a study by Zhang et al. [20], eye movements and cognitive load was recorded in participants as they searched for target items presented in vertical format, block format, and T-format. Participants then were asked to answer a questionnaire about the logic of product information format after they had completed the search task using a given format. Eye movements were found to be smoothest when processing the vertical format and information was processed more efficiently as compared to the block format. Moreover, participants perceived the vertical format to be more logical than the block format. Further support for the vertical format comes from studies by Laarni et al. [21] and Rayner et al. [22], who found that participants required only one fixation point per new line when reading a vertical list of words. They also found that participants’ fixation point was slightly to the left of the center of a word. In another study, the effect of list layout on search speed and eye movements was examined. There were fewer fixations and smaller saccade amplitudes when processing vertical than horizontal lists of the same length. However, search times were not affected by list orientation due to the longer fixation times required for vertical lists [23]. Conclusively, in practice, no extent research appears to suggest a clear differential benefit in terms of using either rows or columns [24].

## 2. Methods

### 2.1. Material and Participants

The Cantonal Police of Zurich provided 593 scans of completed, anonymized checklists. The information was manually transferred to Microsoft Excel and coded considering the goals of the statistical analyses, which were to evaluate the reliability of the checklist for measuring the construct ‘driving impairment’ and to group items according to their similarity following the result of a hierarchical cluster analysis. Coding involved dichotomizing each relevant item by labeling the item as 1 if the observation of the driver was considered normal and 0 if it was considered an indication of impairment. For instance, if the driver showed no signs of physical conspicuity, meaning that they seemed fit to drive, the item was coded with a 1. Conversely, if physical conspicuities were observed, e.g., if the driver was sweating, trembling, restless, or vomiting, then the item was coded with a 0. In terms of lighting conditions, daylight was considered normal and labeled with a 1, while all other lighting conditions were labeled with a 0. The open questions were evaluated via the categorization of reported information. Pupil size estimation was not included in the analyses, as both a very large and a very small pupil size can indicate impairment, and information regarding whether the pupil size was considered unusual was not available. In addition, the item regarding the driver’s behavior during the official act did not relate to the assessment, as it was recorded after impairment had been detected, that is, when police officers left the drivers after bringing them to the place where the blood sample was taken (e.g., hospital).

To evaluate the redesigned checklist, 11 laypeople participants (8 f, 3 m, mean age = 31 years, SD = 12 years) and a similarly aged group of 13 police officers (4 f, 9 m; mean age = 32 years, SD = 4 years) were interviewed. The laypeople were recruited via social media and the police officers were recruited by the traffic police of Zurich.

### 2.2. Procedure

The redesign of the original checklist (Figure 1) involved five steps. In the first step, a reliability analysis based on the Kuder–Richardson formula (KR 20), which is identical to the Cronbach’s Alpha for dichotomous data, was performed. To reduce the quantity of information, the synonymy of response categories within items was checked using the dictionary of the Standard High German language [25]. Alongside this, a frequency analysis was conducted to identify the response categories, which were rarely observed. This information was used in combination with the results of the reliability and synonymy analyses in arguing for excluding response categories with low relevance. In the second step, after the removal of the information identified in the first step, a hierarchical cluster analysis was applied to the 593 datasets to assess the similarity of the items and group them accordingly to improve clarity. In the third step, observations listed in the open questions were categorized, and answers reported were included as additional items in the checklist if they were observed more frequently than the least frequent 25% of the reported response categories in the original checklist. The fourth step involved the application of the design criteria for checklists reported in the literature. The item pool resulting from the previous steps (removed items following the reliability, synonymy, and frequency analyses, and added frequently reported conspicuous observations that were not included in the original checklist) was used after considering results of the cluster analysis from the third step. Discussion of the way in which the elements of the checklist (i.e., checkboxes, items, response categories, pupil template, and open questions) should be arranged ensued. Finally, the redesigned checklist was evaluated in the fifth step by interviewing both laypeople and the police officers using the checklist in their work. Participants were presented with both the original and the redesigned checklists via an online survey. Both laypeople and police officers were asked to rank the two checklists based on their subjectively perceived visual complexity and to explain the reason for their rankings. Laypeople, whose perception of visual complexity of the checklists was assumed not to be influenced by any experience with the checklist, were included in this evaluation to avoid familiarity bias on the part of police officers. A more detailed procedure was adopted for the individual interviews of the police officers; the interviews were semi-structured and notes were taken throughout. The interviews were conducted by one of the authors and took approximately 25 min each. Before each interview began, the goal of the interview process was briefly explained. The interviews are considered to be of high quality for several reasons: Firstly, it was expected that the police officers answered honestly, because the data were recorded anonymously. Secondly, the interviewees had the ability to formulate and give concise answers and provide precise explanations for them based on their experience with using the checklist. Thirdly, the interviewer used a guide to reduce the risk of compromising the validity of the recorded data. The guide was used flexibly, meaning that questions were asked in no predefined order and mentions not included in the questions could be added during the interview. The interviewees were asked questions that compared the two checklists as well as open questions related to the design and content of the redesigned checklist. Predefined questions were prepared based on the feedback questionnaire used by Pucher et al. [26] on the subjective perception of checklists and the system usability scale [27]. Police officers were also asked about appropriateness of the font, font size, distances between elements, and whether they agreed with the new pool of response categories.

The fifth step included an attempt to evaluate the improvement quantitatively by measuring the total path lengths for filling out the checklists, computing the total ID for filling out the checklist by hand, and defining the visual complexity. The path length was measured by connecting all of the elements that the police officers could respond to. For the computation of the total ID [17], the measured width of each checkbox (0.2 cm) and the amplitude of the pointing movement from one to the next was considered. All IDs were summed up to a total ID. For the sake of simplicity, for the elements to which the users respond by writing down their observation on a line (e.g., pupil size), the start of the line was chosen as the target location having the same width as the checkboxes (0.20 cm). To quantify improvements in visual complexity, Tullis’s [28] procedure was adopted, and the visual complexities of the original and redesigned checklists were compared.

### 2.3. Data Analysis

Reliability and hierarchical cluster analyses were performed using SPSS version 26.0 (IBM Corp., Armonk, NY, USA, 2019). Reliability was evaluated by calculating the Cronbach’s Alpha of the data. For the hierarchical cluster analysis, the proximity measure Jaccard (asymmetric measure—impaired more important than not impaired) and the cluster method completed linkage were used. The item frequency analysis was conducted using Excel (Microsoft, Redmond, WA, USA, Version 2102).

## 3. Results and Discussion

### 3.1. Step 1: Reliability, Frequency Analysis, and Synonymy

The results of the reliability analysis considering 15 items of the checklist are listed in columns 2 and 3 of Table 1. The analysis revealed a Cronbach’s Alpha of 0.58. According to Vaske et al. [29], a Cronbach’s Alpha of between 0.65 and 0.80 indicates an adequate internal consistency. The reliability may be improved by removing items with a weak or negative correlation, after which the Cronbach’s Alpha increases significantly [29,30]. Following the results reported in columns 2 and 3 of Table 1, the item ‘Lighting’ (lighting conditions at the control site) showed a negative correlation and was removed in the redesigned checklist. A negative correlation was also found for the item ‘Cannabis odor’. A discussion with police officers who used the checklist revealed that they had been instructed to process the items ‘Alcohol odor’ and ‘Cannabis odor’ only if the consumption of alcohol or cannabis was suspected to be the cause of driving impairment. These two items were processed a posteriori, not alongside the other items, and were therefore excluded from the analysis. A weak correlation was also found for the item ‘Command of German’. This item was deleted because it is not directly related to impaired driving. When considering only the 11 remaining items, the reliability of the checklist increased; the new Cronbach’s Alpha was 0.67. As shown in columns 4 and 5 of Table 1, ‘Pupil light reaction’ still showed a weak correlation (0.073), which could point to a difficulty in estimating abnormal behavior in this regard. However, a deletion of this item would not significantly increase the Cronbach’s Alpha. Following clarifying conversations with police experts, the items ‘Alcohol odor’, ‘Cannabis odor’, ‘Pupil light reaction’, and ‘Command of German’ were deemed relevant to other aspects of traffic stops and, therefore, could not be excluded from the checklist.

Table 2 below lists the frequencies for the reported response categories. The least 25% of response categories were reported with a frequency of 7.93% or less (Table 2). The categories ‘Appearance: neglected’, ‘Physical signs: vomiting’, ‘Response: arousable’, and ‘Response: sleeping/unconscious’ were observed in less than 2% of the cases. As this quantity is negligible, they should be excluded from the checklist. Moreover, the analysis of synonymy revealed redundant response categories. The German words for mumbling and slurred speech are very similar in meaning [25]; as such, it is likely that police officers have difficulty differentiating between those adjectives and therefore ‘Speech: mumbling’ should be excluded from the checklist. Additionally, as according to the dictionary of the German language, the German translations of unsteady and staggering are synonyms [25], ‘Gait: staggering’ should be excluded from the checklist, as its frequency of occurrence was lower (2.87%, Table 2) than that of the category ‘Gait: unsteady’ (>9.28%). Furthermore, as aggressive and provocative are synonyms, discarding either one or the other is recommended; as provocative behavior was reported more frequently, ‘Mood/Behavior: aggressive’ should be excluded from the checklist. The items ‘Reaction: extremely delayed’ and ‘Reaction: delayed’ were also consolidated, as this difference in time is not quantified. Lastly, it is likely that holding on to the vehicle is a result of being unbalanced. As the item ‘Getting out of the car’ includes an item ‘unbalanced’, ‘Getting out of the car: has to hold on to the car’ was excluded from the checklist.

Furthermore, pupil sizes were reported in 565 out of 593 VERIFY cases; of those, there were only 20 in which different sizes were reported for the right and left eyes, making it reasonable to ask for only one.

### 3.2. Step 2: Grouping of Similar Items Using Hierarchical Cluster Analysis

**Figure 2:**
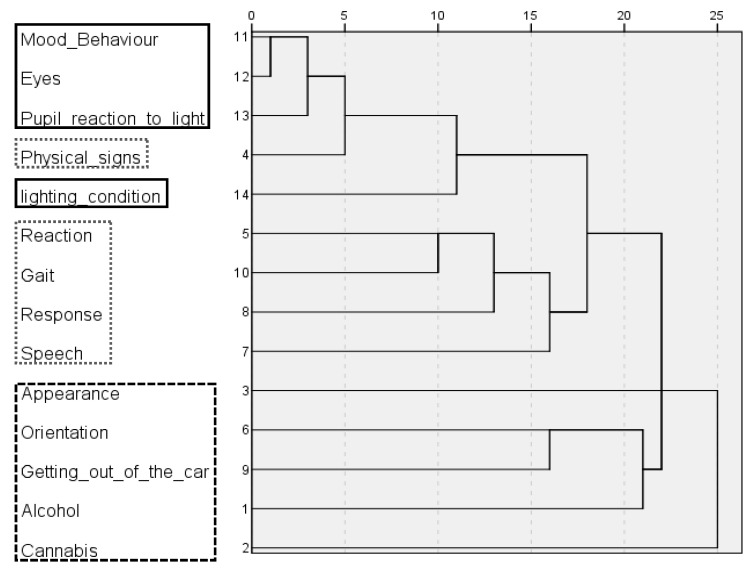
Dendrogram obtained by cluster analysis of the checklist items. Lines outline three groups of items, each of which forms a column in the redesigned VERIFY checklist (solid black line: first column; grey dashed line: second column; dashed black line: third column).

As explained in the introduction [8], checklists should be divided into smaller parts, each with items addressing similar functions, and the most critical items should be placed as close as possible to the beginning. Following this rule, elements that are relevant to driver impairment assessment at the time of traffic stop were put at the top of the redesigned checklist and separated from the other items (e.g., command of the German language) by the horizontally arranged pupil size template. The checklist items above the pupil size template were arranged into subparts. For the sake of visual simplicity, only three subparts were defined. A hierarchical cluster analysis searching for a three cluster solution was run to quantify the similarity of the items. The resulting dendrogram (Figure 2) revealed which impairments described by the items were jointly observed and which were not. The dendrogram indicated that items of the categories ‘Mood/Behavior’, ‘Eyes’, ‘Pupil reaction to light’, ‘Physical signs’, and ‘Lighting condition’ were often marked off, and the five items should be put in the same subgroup. Due to the lack of space, ‘Physical signs’ was moved to the second column. Although ‘Physical signs’ was more similar to ‘Eyes’, ‘Mood/Behavior’, and ‘Pupil reaction to light’ than ‘Lighting condition’, it was moved to the second column, because it makes more sense to group ‘Lighting condition’, which is important for observing eye conditions, with other items related to the eyes. The second and third column were formed exactly according to the cluster analysis.

### 3.3. Step 3: Categorization of the Text from the Open Questions

Observations that were frequently reported in the open questions are listed in Table 3. It is worth noting that the added items could contribute to improve the reliability of the checklist and its effectiveness in detecting impaired drivers. In approximately one-fifth of all cases, the driver was found to be behaving nervously. Nervousness can be an effect of drugs, as the work of Baylen and Rosenberg [31] showed. It is therefore suggested to create a category entitled ‘Mood/Behavior: nervous’. Additionally, a dry mouth was observed in almost 20% of the cases. Dry mouth has been shown to be a consequence of drug ingestion in several studies [32,33]; hence, consideration should be given to including the response category ‘dry mouth’ in the ‘Appearance’ item of the checklist. In 13% of the cases, the police officers observed no pupil reaction to light. A slow pupil reaction, or lack thereof, has been reported in the literature [34,35] as a sign of THC consumption and, thus, a new response category entitled ‘no reaction to light’ for the item ‘Pupils’ is recommended. Furthermore, approximately 10% of the police officers mentioned the extent to which the driver followed their instructions (e.g., opening the window, turning off the radio). Since observing how drivers cope with multiple tasks belongs to a key part of the VERIFY procedure [9], it would make sense to add ‘Completion of tasks: yes/partly/no’ to the checklist. In approximately 8% of the cases, a white-coated tongue was observed; this can occur after THC consumption. In a study of physiological signs of drivers who had consumed THC, this tongue coating was found to be a reliable indicator of the presence of THC [36]. An additional response category ‘white tongue’ should therefore be added to the item ‘Appearance’.

### 3.4. Step 4: Redesigning the Checklist

The new pool of items resulting from the reliability, frequency, and synonymy analyses, the categorization of open questions, as well as the pupil template, the two open questions, and the three items ‘Behavior during official act’, ‘Command of German language’, and ‘Duration of the official act’ were used in the redesign of the checklist. The items above the pupil template were distributed into the three subgroups according to their similarity in accordance with the results of the hierarchical cluster analysis. The item ‘Pupil ⌀’ and the new item “Followed instructions”, which were not included in the cluster analysis, were added to the checklist by logical or structural considerations. The pupil size was added to the first column to the group of items on eye observation directly above the pupil size template. The item “Followed instructions” was added to the end of the third column, as there was sufficient space.

Subsequently, the question of whether the checkboxes should be placed to the left or right of the label was discussed. As the word identification span is only three to four characters to the left but 14–15 characters to the right of the fixation [22], placing the checkbox on the left side of the label better supports visual information processing. An analysis of the original checklist, shown in Figure 1, revealed a median number of characters per label of ten. By placing the box to the left of the label, more than 50% of the items could be processed while fixating on the box.

**Figure 3:**
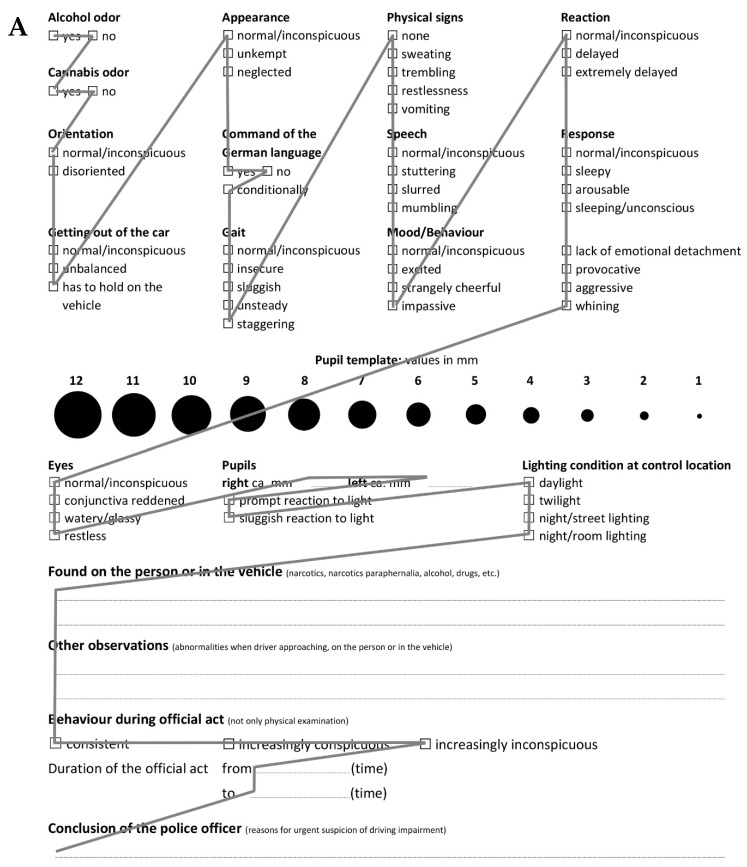

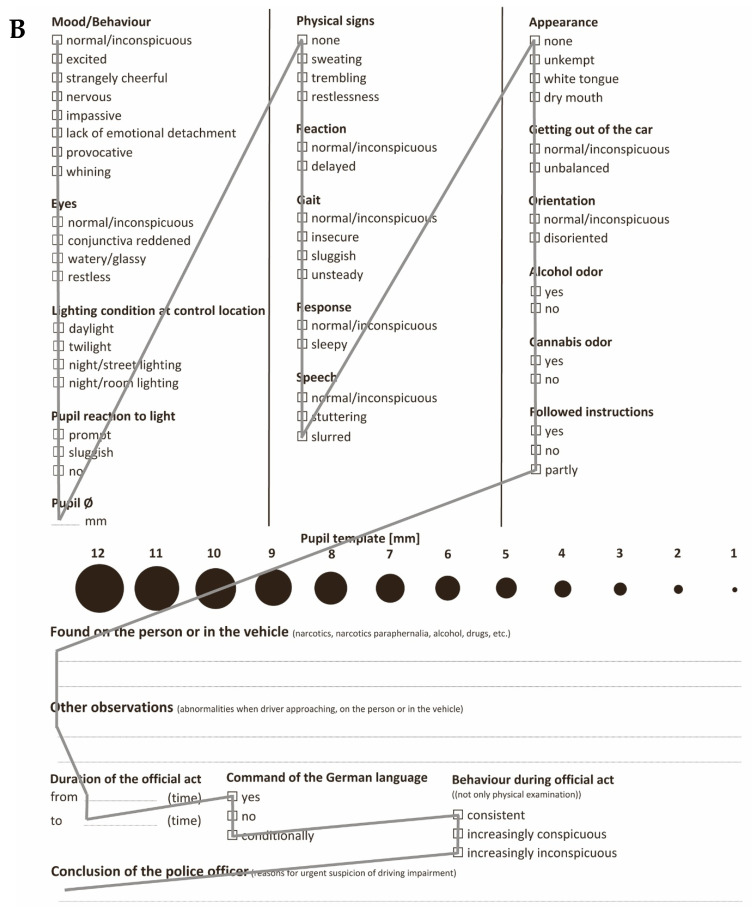
Grey lines represent movement amplitude in the original (**A**) and redesigned (**B**) checklists.

Next, the arrangement of the subgroups and the organization within each subgroup was evaluated. As the path travelled during a pointing movement determines the duration of that movement, the distance from one box to the next was used to evaluate the vertical and horizontal arrangements of the checkboxes. The total path taken when filling out the checklist depends on the number of characters in the labels separating two checkboxes horizontally, the distance between subsequent rows, and the direction moved during the processing of the checkboxes. Considering that the checkbox has a width of one character and the median size of the label is ten characters, the travel path from one horizontal checkbox to the next is 11 characters. When considering arranging the rows with an optimal line spacing for reading text (e.g., [37]), the vertical separation of two checkboxes is approximately 120–150% of the character height. As character heights are approximately 1.5 times their widths, we expect that processing checkboxes from top to bottom, column by column, is more efficient compared to processing them row by row and top to bottom. When measuring the paths for filling out both the original and redesigned checklists (Figure 3), the vertical processing of the checkboxes was found to be the better strategy.

**Figure 4:**
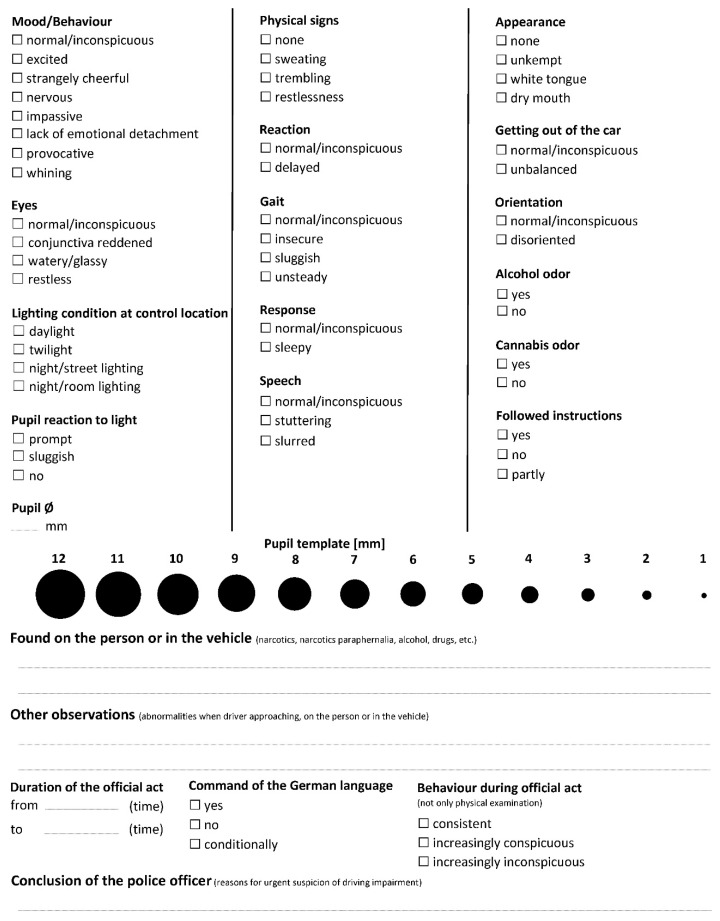
The redesigned checklist.

Visual complexity depends on the variety of the visual qualities in a displayed object; as such, the number of different objects in the redesign was minimized as much as possible. The same font was used throughout the checklist, and the titles were bolded to clarify changes in items. A clear visual hierarchy was established by separating columns by vertical lines; in this way, users are guided along a path, which reinforces the column-by-column completion of the checklist. White spaces were used to separate the subgroups, while response categories within an item were placed close to each other. Disordered information also enhances visual complexity, meaning that information should be organized by aligning items and response categories within columns and rows. The redesigned checklist is presented in Figure 4.

### 3.5. Step 5: Evaluation of the Redesigned Checklist

The appearance of the new protocol was found to be simpler than the original protocol by all 11 laypeople and by 12 out of 13 police officers. Among the reasons given for this opinion were the vertical lines, which made the document more structured and clearer. Only one of the interviewed police officers found the appearance of the original protocol to be simpler, because it was what they were used to.

The responses of the police officers to the predefined questions are presented in Table 4. Police officers were asked to answer the questions by comparing the redesigned checklist to the original. As each question regarding the redesigned checklist was answered positively by at least 78% of the police officers, it can be assumed that the redesigned checklist was seen as an improvement over the original.

In the interviews, the police officers stated that the new structure could lead to fewer instances of forgetting to check a box when going through the protocol, as the vertical lines give a clear direction. Another improvement mentioned was that ‘Mood/Behavior’ was in one column, because this way the affiliation of the response categories to this item was clearer. Respondents also liked the fact that ‘Duration of official act’, ‘Command of the German language’, and ‘Behavior during official act’ were now separated from the other categories, as they do not directly contribute to determining whether someone should be assessed as impaired to drive. Three respondents suggested improving the effectivity of the checklist by adding space under each item that could be used to report additional response categories. All respondents agreed with the appropriateness of the font size, font type, and spacing between the individual elements. For them, the most important thing was that everything is listed on one page. However, as far as the application of the redesigned checklist in practice was concerned, three police officers believed that completing the checklist had not been significantly simplified, even though they stated that the redesigned checklist looked simpler than the original one. The rest of the respondents believed that completing the redesigned checklist might be easier in practice, particularly for those unfamiliar with the original.

The following results show whether excluded and included response categories complied with the experience of the police officers. Police officers responded positively to the removal of the following items (rate of positive responses are given in percentage): vomiting (100%), sleeping/unconscious (92%), arousable (84%), neglected (62%), staggering (15%), mumbling (23%), aggressive (15%), extremely delayed reaction (15%), and has to hold on the vehicle (8%). In terms of the additions, they responded positively as follows: white-coated tongue (84%), dry mouth (84%), following the instructions of the police officer (61%), no pupil reaction to light (100%), nervous (54%), and combining the left and right pupil size to one response category (92%).

The improvement in the redesigned checklist was further quantified by computing the total path length required for completing the checklist, the Fitts’s ID, and the visual complexity. When measuring the paths for filling out the two checklists (Figure 3), the total distance covered was approximately 120 cm in the original and 79 cm in the redesigned version for the same magnification factor of the A4 size checklist, resulting in a difference of 41 cm and reducing the travel path by approximately 34%. In addition to different path lengths, the two different processing strategies varied in degree of difficulty in performing the pointing movements. The sum over all indices of difficulty was 164 bits for the original and 135 bits for the redesigned checklist, meaning that the redesign reduced the level of difficulty in processing the checklist by approximately 18%. The quantification of visual complexity, as per Tullis [28], resulted in a complexity of 381 bits for the original and 298 bits for the improved version, a reduction of approximately 22%.

## 4. General Discussion

In this study, we redesigned a checklist for detecting impaired drivers. In the development of the original, human factors and ergonomics principles, particularly Gestalt principles, the horizontal/vertical arrangement of the items, reading direction, visual complexity, and Fitts’s law regarding pointing tasks were not considered; as such, there was a potential for improvement.

The checklist was redesigned and optimized using a set of 593 checklists recorded during real traffic stops. Some response categories were removed, and some new ones were added; in total, the number of elements processed by the police officers was reduced from 65 to 62. A reduction of just three elements is small, but the new pool of elements better complies with their needs in practice. An even better optimization was possible, but compromises were required. For instance, items regarding alcohol and cannabis odor were kept despite the results of our analysis. There were two reasons for this: Firstly, the consumption of illegal drugs that affect driving skills, such as cannabis, is becoming more prevalent and the police are concerned with crime detection in general. In terms of alcohol, alcohol consumption is one of the most common causes for severe accidents in Switzerland [38]. As such, in addition to preventing potential accidents, checking for alcohol odor has the potential to incentivize drivers to refrain from drinking and driving, in addition to the effect of educational prevention programs such as the ‘You Drink, You Drive, You Lose’ program [39].

In addition to creating a more suitable item pool for driver impairment assessment, the elements of the redesigned checklist were organized more logically than those in the original. The most relevant elements were put at the top of the list and were clearly separated from the less relevant ones, such as ‘Command of the German language’, by the pupil size template. The arrangement of the items has also improved; in the original checklist, the response categories of the item ‘Mood/Behavior’ were split into two columns, which caused confusion, especially when the checklist was completed column-by-column, because there was no title above the response categories for the item in the second column. In the redesigned checklist, all response categories for a given item are arranged in the same column with equal spacing between the categories, which is in accordance with findings of Zhang et al. (2017) and meets the Gestalt principle of proximity.

In an attempt to quantify improvement in the redesigned checklist over the original, total path lengths for filling the checklist, the Fitts’s index of difficulty, and the visual complexity of both were computed. Imposing a processing of the items column by column by adding vertical lines resulted in a total path length that was 34% shorter in the redesigned protocol than in the original. In addition, the redesign led to an ID reduction of 18%. The vertical lines also simplify completion, as users do not have to orient themselves first. Without vertical lines, it is more likely that users will complete the checklist in an order other than that presented in Figure 3, resulting in an even stronger increase in total path length. Finally, computed visual complexity was reduced by 22%. Overall, our results indicate that the redesign improved the efficiency of the checklist. This improvement in visual complexity is consistent with the subjective complexity assessment of both laypeople and police officers. They stated that the redesigned protocol looked easier than the original, which is likely due to the improvement in the visual hierarchy due to the separation of the columns by lines. As new relevant items for the detection of driver impairment were included, the redesign is capable of being more effective than the original. For more accurate quantitative statements, an additional study in which completion times and failures to complete the checklist are recorded would be necessary. Moreover, for more precise qualitative statements regarding the significance of the improvements of the usability of the checklist, the redesigned checklist should be evaluated over a longer period of application. In this way, users’ behavior, performance, and satisfaction will be measured in a natural environment to uncover problems and opportunities in the design.

## 5. Conclusions

This work presents the redesign of a checklist used in the detection of driving impairment. The redesign led to a more efficient and effective checklist. This improvement is of particular importance as it helps account for various factors that interfere with the accurate processing of checklists during traffic stops. Some of the most relevant include the potential completion of the checklist at an elevated level of stress or within a limited time, and the fact that the result of the driving impairment detection could have severe consequences for both the driver and the police officer. One general conclusion drawn from this study is that the need for compromises when applying ergonomics in practice interferes with purely science-based optimization, resulting in a potential reduction in the quality of the studied product. Solutions to account for this should be found through a more holistic redesign approach.

As a next step, the findings will be evaluated over a longer period of application of the redesigned checklist in practice.

## Figures and Tables

**Table 1:** Reliability analysis of the original checklist, including 15 items (columns 2 and 3), and of the reduced checklist after the exclusion of four items (columns 4 and 5). Pupil size estimation is not included. Cronbach’s Alpha—original checklist, 0.58; reduced checklist, 0.67. Number of checklists processed = 593.

Items (Observer Non-Conspicuity)	Original Checklist (15 Items)		Reduced Checklist (11 Items)	
	Corrected Item Total Correlation	Cronbach’s Alpha if Item Deleted	Corrected Item Total Correlation	Cronbach’s Alpha if Item Deleted
Alcohol odor	0.123	0.581		
Cannabis odor	−0.126	0.628		
Appearance	0.286	0.552	0.089	0.652
Physical signs	0.112	0.583	0.114	0.673
Reaction	0.439	0.517	0.260	0.619
Orientation	0.366	0.537	0.217	0.629
Command of German	0.080	0.584		
Speech	0.323	0.543	0.137	0.639
Response	0.399	0.526	0.256	0.625
Getting out of car	0.394	0.531	0.273	0.629
Gait	0.452	0.513	0.318	0.611
Mood/Behavior	0.272	0.556	0.130	0.651
Eye	0.160	0.574	0.081	0.669
Pupil light reaction	0.062	0.586	0.073	0.677
Lighting (daylight)	−0.054	0.617		

**Table 2:** Frequencies of rarely reported response categories.

Response Categories—Original Checklist	Frequency (%)
Physical signs: vomiting	0.00
Response: sleeping/unconscious	0.51
Appearance: neglected	1.01
Response: arousable	1.52
Gait: staggering	2.87
Speech: mumbling	4.55
Mood/Behavior: aggressive	5.23
Reaction: extremely delayed	6.41
Getting out of the car: has to hold on to the vehicle	7.93
Mood/Behavior: provocative	7.93

**Table 3:** Frequencies of the most reported items found in the open questions.

Items Reported in the Open Questions	Frequency (%)
White tongue	8.43
Failure to follow the police officer’s instructions	10.12
No pupil reaction to light	13.32
Dry mouth	19.22
Nervous behavior	21.92

**Table 4:** Police officers’ responses to questions comparing the redesigned checklist withthe original.

Question	Positive Response
The checklist looks simpler	92%
I think I would like to use this system frequently	92%
The checklist is easier to use	78%
The checklist covers conditions relevant to my practice	100%
I find this checklist applicable to my practice	100%
I think I would prefer the new designed protocol	92%
I find the system unnecessarily more complex	0%
I find the system more cumbersome	8%

## Data Availability

The data presented in this study are available on request from the corresponding author.
